# Design, Implementation and Evaluation of an Emotional Education Program: Effects on Academic Performance

**DOI:** 10.3389/fpsyg.2020.01100

**Published:** 2020-05-28

**Authors:** María-José Mira-Galvañ, Raquel Gilar-Corbi

**Affiliations:** Departament of Developmental Psychology and Didactics, University of Alicante, Alicante, Spain

**Keywords:** emotional education, academic performance, primary education, cooperative learning, quasi-experimental design

## Abstract

**Background:** In recent decades, the amount of research on social and emotional learning programs in schools has increased significantly, showing a great number of positive student outcomes, including greater ability to perceive, understand and manage emotions, better attitudes about self and others, less aggressive and/or disruptive behavior, higher levels of psychological well-being and improvement in academic performance among others. The purpose of this research was the design and implementation of the OKAPI emotional education program. A multidimensional program based on cooperative learning methodology.

**Methods:** 86 students of Primary Education, from 3rd to 5th grade (45 students in the experimental unit and 41 in the control group).

**Results:** The implementation of the OKAPI has a positive impact on academic achievement.

**Conclusion:** This program shows the convenience of incorporating programs that can be integrated into school life and can be applied by the teaching staff using both social emotional learning and school climate approaches.

## Introduction

Emotional education programs have been related, in the last decades, not only to the improvement of social competences such as the ability to perceive, manage or understand emotions (Extremera and Fernández-Berrocal, 2004; Lópes and Salovey, 2004; Brackett et al., 2006), but also with better results in academic performance (Petrides et al., 2004; Gil-Olarte et al., 2005; Palomera and Brackett, 2006; Collaborative for Academic Social and Emotional Learning [CASEL], 2007; Pérez and Castejón, 2007; Nasir and Masrur, 2010; Fernández-Berrocal et al., 2012), the increase in positive behaviors (Mestre et al., 2006), and higher levels of students’ self-esteem (Salovey et al., 2002; Fernández-Berrocal et al., 2006), among others.

Unfortunately, the implementation of some social and emotional programs has not always yielded the expected results. This is due to the fact that the programs have not been fully incorporated into the school routine, or they are based only on direct instruction and without full and continuous support for teachers and school staff (Durlak et al., 2011; Jones and Bouffard, 2012). There are bidirectional relations between children and contexts in which they learn and nurture their social, emotional and academic competence (Bronfenbrenner and Ceci, 1994). Students’ social-emotional development is closely linked to the environment in which they develop. A positive school climate is required in order to support student learning and provide opportunities for social and emotional development. Both aspects have health benefits and a positive impact on students’ life (Osher et al., 2014; Greenberg et al., 2017).

School climate and Social Emotional Learning are essential components of effective schools (Osher et al., 2004). This trends shows that the most effective programs create safe conditions to practice the skills learnt in the program, engage multiple members of the school community, provide professional development for teachers and school staff. Furthermore, teachers are in charge of their application. The current research seeks to prove the effectiveness of the OKAPI program, a multidimensional program that uses both school climate and social and emotional learning approaches and also incorporates active methodologies, such as cooperative learning, to encourage interaction among students.

### Emotional Education

Emotional education is a continuous and permanent educational process that aims to promote emotional development as a complement to the cognitive development of the child. Emotions can facilitate or impede children’s academic engagement and achievement (Zins et al., 2004; Gil-Olarte et al., 2006; Pérez and Castejón, 2007; Durlak et al., 2011; Blake et al., 2015). Effective mastery of social-emotional competences is associated with greater well-being and pro-social behavior (Greenberg et al., 2003). It can also reduce risk factors and prevent certain dysfunctions (Bisquerra, 2000, 2003).

According to Collaborative for Academic Social and Emotional Learning [CASEL] (2007), social and emotional learning programs (SEL) can be defined as the capacity to recognize and manage emotions, solve problems efficiently, and establish positive relationships with others. CASEL establishes five different competencies that reflect both intrapersonal and interpersonal spheres: self-awareness (the ability to recognize and comprehend one’s own emotions, personal goals and values), self-management (the ability to regulate emotions and behaviors), social awareness (the ability to understand the perspective of others, no matter their cultural background), relationship skills (the ability to establish positive relationships) and responsible decision making.

In the past two decades, research on emotional education has shown that the most effective programs are the ones that deliver explicit lessons that teach social and emotional skills, provide opportunities for students to use those competences throughout the day (Weissberg et al., 2015) and have multi-year and multicomponent approaches (Zins et al., 2004).

In addition, Durlak et al. (2011) found that programs are likely to be effective if they are taught by teachers, involve the school community and are also evaluated during the process and at the end of the program. They also need to follow step-by-step SAFE criteria (sequenced, active, focused, and explicit): use a sequenced training approach, use active forms of learning, focus sufficient time on skill development, and have explicit learning goals (Durlak, 1997; Bond and Hauf, 2004).

There are three main ways of socializing emotions: through direct exposure and observation; making use of direct teaching and/or coaching and fostering opportunities for practice (Brenner and Salovey, 1997). That is why it is crucial teachers’ emotional training. Teachers with high EI scores generate a more positive school climate (Extremera et al., 2003; Brackett et al., 2010) which helps the program implementation and gives more opportunities to practice the socials and emotional skills.

### Cooperative Learning

From the perspective of social relationships, there are three different types of educational situations that take place in the classroom: individualistic, cooperative and competitive (Pujolàs, 2001; Johnson and Johnson, 2009; Arnaiz and Linares, 2010; Vega and Hederich, 2015). Students who work in a cooperative way can have higher levels of achievement because the collective production is higher in quantity and quality than individual production. It also allows students to develop their emotional intelligence through numerous socials interaction (Bisquerra et al., 2015) so they can develop social and interpersonal skills while they are working in teams (Díaz-Aguado, 2003; Linares, 2004).

The OKAPI program is based on a cooperative learning methodology. We define cooperative learning as: “a continuum of learners working together in a small group, so that everyone can participate in the collective task that has been clearly defined by the teacher. So students work together to learn and are responsible for one another’s learning as well as their own” (Slavin, 1995).

In cooperative learning, students work in small and heterogeneous groups. It involves students working together to achieve common goals and requires the contribution of all group members in order to fulfill individual and group objectives. Students are responsible for their own learning process and, at the same time, are responsible of their peers’ learning process. Peer interaction and peer tutoring are promoted. Knowledge is built collectively taking into account the classroom environment and social relationships.

Cooperative learning specifically contributes to the development of interpersonal intelligence, because the skills needed for social interaction are practiced on a daily basis and developed in an indirect way (Linares, 2004). In fact, there are important similarities between cooperative learning and training in social skills (León et al., 2014; Larraz et al., 2017). In cooperative learning students learn social skills through modeling, they practice those skills in real situations and they receive feedback concerning their behavior from their classmates (León et al., 2015). But the necessary socials skills for effective cooperative work need to be learned beforehand (Gillies and Haynes, 2011). So cooperative learning and social emotional programs are interdependent.

Over the past decade, cooperative learning has emerged as an one of the most effective approaches to classroom instruction. According to research, students who using cooperative learning methodology improve their academic achievement, have a greater number of positive social-skills and prosocial behavior, higher self-esteem and better motivation (Ovejero, 1993; Johnson et al., 1999; Pujolàs, 2001, 2005; Díaz-Aguado, 2003; Wilson-Jones and Caston, 2004).

### OKAPI

The OKAPI Learning Environment program aims to create a positive classroom and school climate that enables the development of the socio-emotional competencies of all members of the educational community.

The program uses direct instruction to teach socio-emotional skills and it has different tools that are applied cross-sectionally and are incorporated into everyday routines. It also favors the creation of a positive environment where students, through interactions within the particular context, put into practice the socio-emotional competences. In order to implement the program, each class had to incorporate in their schedule 10 to 15 min during the morning to do an assembly, approximately 5 min to practice full attention after the recess and 30 min per week (3 times per month) to work in different activities related to the program.

The key components of the program respond to the Catalan acronym of OKAPI: O (Classroom Organization: routines, rules, operation, distribution of spaces, etc.), K (Cooperative Learning as a main methodology for the teaching-learning process) A (Positive classroom environment), P (Participation: Involvement of all members of the educational community) and I (Emotional intelligence: emotional education as a key point in the curriculum).

The main characteristics of the program can be summarized as follows:

#### Cooperative Learning

Cooperative learning has been used as a tool to improve the classroom climate. Every month, students do teambuilding activities and class building activities. These are specifically designed to stimulate peer interactions, promote social cohesion and to improve social skills. Cooperative learning was the main methodology used in the different subjects and it represents a basic tool for the program. Several cooperative learning strategies were used in the program such as Quiz, Quiz trade, Round Table or Rally Coach (Kagan, 1994) among others.

#### Positive Classroom Environment

Three aspects are emphasized to improve the classroom environment explicitly. First, effective and coherent plans to develop positive behaviors in a specific class and in the school. Second, strategies for conflict resolution: The VET technique (evaluates, explores, and transforms) to be used by teachers. And students put into practice the PiP technique (tool that encourages students to stop and think when they have a problem using questions to reflect on the situation) and MENU (tool to find strategies that guide students how to resolve conflicts with other people). Finally, the improvement of communication between families and schools using different strategies (student of the month, weekly self-report card, etc.).

#### Emotional Intelligence

Emotional competencies are taught through direct instruction, approximately two times per month in a 30 min class using the Bisquerra (2009) five block model: emotional conscience, emotional regulation, emotional autonomy, social competency, life skills and well-being. There were seven sessions of direct instruction. Also, after every recess, students perform full attention techniques and strategies for about 6 to 7 min (overall, at least 30 min per week).

Moreover, in the class schedule there is 10 to 15 min for classroom assembly, named in the OKAPI program as a “Start Circle,” at the beginning of the day. In the Start Circle the teacher explains the planning of the day, students work with the mood meter (a technique used to label, manage and understand emotions) and it is a time where students can talk and share their thoughts and feelings. Also, every Friday during the “Start Circle,” one word pertaining to emotions is introduced. The emotion is explained by the teacher, discussed in the assembly and associated to real situations. Students discuss that word with their families during the weekend and the following Monday, during the assembly it is explained again and introduced onto the mood meter.

Furthermore, in the language areas we use the strategy CiC (Tell and Share). After reading some stories, students, using a cooperative learning strategy, discuss the feelings of the character, consequences of their decisions and look for different alternatives.

### Research Questions

This study seeks to prove the effectiveness of an emotional education and positive climate program based on cooperative learning methodology for primary school students. The purpose of this research is to analyze the contribution of the OKAPI program on academic achievement and the improvement of emotional competence in students.

The questions that guide this work are as follows: Can an emotional educational and positive climate program based on cooperative learning such as the OKAPI program improve the academic performance of students?

We hypothesize that we will observe statistically significant differences in academic performance and in social competence between the experimental and the control group.

## Methods

### Participants

A non-probability convenience sampling was used to select the participants. Two different schools from Alicante Province participated in the current study. The selection of these schools were based on knowledge of the characteristics of both schools as well as their populations, and their willingness to participate in the study. Both schools have similar characteristics: they are public schools, they have a similar size and they use the same textbooks. The total sample was composed of 86 students from two different primary schools.

Students from 3rd grade (9 to 10 years old), 4th grade (10 to 11 years old) and 5th grade (11 to 12 years old) participated in the current study. Overall, six different classrooms: three in the control group and three in the experimental group.

The experimental group consisted of 45 students: 15 from the third grade, 14 from the fourth grade and 16 from the fifth grade. In the control group there were 41 students: 14 from the third grade, 14 from the fourth grade and 13 from the fifth grade. In total, 61.7% were boys and 38.3% were girls.

### Instruments

The following instruments were utilized in this study:

(1) Emotional Quotient Inventory: Young Version (EQ-i:YV) (Bar-On and Parker, 2000). This questionnaire measures the level of emotional and social functioning in children and adolescents providing an estimate of their underlying emotional and social intelligence. It consists of 30 items with values on a 5-point Likert-type scale and it evaluates five general factors of EI. The EQ-i:YV (s) has adequate internal reliability in every dimension: interpersonal scale (*a* = 0.67), intrapersonal scale (*a* = 0.84), stress management (*a* = 0.84), adaptability (*a* = 0.83) and the EQ-i total scale (*a* = 0.77). Emotional intelligence was measured through the student’s self-report and through the teacher’s evaluation of each of the students.
(2) Academic performance: This was evaluated using the average grades in the main subjects (Maths, Catalan language, Spanish language, Social science, and Natural science). These scores are the result of assessment tests designed by the teaching staff in accordance with the guidelines established by the competent authority in education.

### Procedure

Prior to the implementation of the study, the researcher was given permission by the principals of each school, as well as teachers and parents of each and every participant in the study. We guaranteed confidentiality relating to all of the data obtained from both teachers and students. This study was carried out in accordance with the recommendations of the University of Alicante Ethics Committee. All participants gave informed written consent in accordance with the Declaration of Helsinki.

Teachers who would participate in the study received training during the previous school year. This training was part of an Innovation project named *Cooperative Learning as a tool to develop emotional competencies.* Teachers received conceptual training in Cooperative Learning, emotional intelligence and OKAPI components in 10 sessions, with a total of 20 h. Moreover, monthly supervision and training sessions during the intervention were implemented to ensure the accuracy and quality of the program.

The quasi experimental study was conducted over a period of 6 months, during a school year. The pre-test was administrated to the experimental and control group in November. The implementation of the Okapi program in the experimental group took place from December to May while the control group received normal classroom instruction. In June, the post-test was administered to both groups.

The OKAPI program was applied by the teachers although the person in charge of the research was in each classroom for at least 3 h per week in order to support teachers and carry out the more complex activities. At the same time, on a monthly basis, training sessions for teachers were conducted and also the teachers carried out self-evaluations as part of the program implementation.

### Design

A quasi-experimental design with non-equivalent control-group and pre-test/post-test was used (Garcia Gallego, 2001). Quasi-experimental design was used because students weren’t randomly assigned to classes by the researcher. The research was conducted over a period of 6 months. The interval between the pre-test and the post-test was 8 months.

### Data Analysis

A robust statistical and methodological treatment based on the principles of the General Linear Model is used to analyze the data (Leon et al., 2003; Ganju, 2004).

The data were analyzed as follows. First, to ensure homogeneity between groups, a comparative analysis was carried out at the beginning of the research. The differences between the experimental and control group were assessed by using Student’s *t*-test. This is used in order to know how significant the differences between the means of two groups are.

Second, to measure the effect of the intervention we used the GLM repeated measure. This statistical technique tests the main effects within and between subjects. Groups of related dependent variables are analyzed which represent different measures of the same attribute (Freeman, 1973; Vuchkov and Solakov, 1980). At least one of the factors is based on independent observations and another one is based on correlated observations. A multivariate variance analysis (MANOVA) and a repeated-measure univariate analysis of variance (ANOVA), in which the measures of dependent variables are treated as variables within subjects (intrasubjects) and groups are treated as variables between subjects, are done (Lavori, 1990; Stemmler, 1994; Keselman et al., 1998; Woodrow, 2014).

A comparison of means was performed to analyze if there were significant differences between dependent variables among control and experimental groups (Duby et al., 1977; Deleon and Atkinson, 1991). Null hypotheses are contrasted in terms of the effects of both intersubject and intrasubject factors, and the interactions between individual effects and factors are investigated (Gabriel and Hopkins, 1974).

Finally, we graphically represent the differences between the experimental and control groups in the pre-test and the post-test situations to interpret the differences. The analysis and graphical representation of the data were performed using SPSS v.20 with a license from the University of Alicante.

## Results

### Initial Situation: Experimental and Control Groups

First, to analyze whether there were differences in EI levels (measured using the EQ-i:YV) in the two groups before the intervention, mean contrast for the independent samples was completed using independent *t* test (continuous-variables). The result showed that there were initial differences in some dimensions of the EQ-i:YV scale answered by the students. Results for the intrapersonal dimension shows differences between control and experimental groups [*t*_(80,74)_ = −2.23, *p* = 0.03]. The control group obtains higher scores than the experimental group. In the stress management dimension there were also initial differences. In this case, the control group also has higher scores [*t*_(84)_ = 3.31, *p* = 0.00]. The EQ-i:YV scale answered by the teachers referring to the students shows differences between the control and the experimental group in the intrapersonal dimension [*t*_(84)_ = 3.84, *p* = 0.00]. So that we can see that the control group has higher scores.

### Analysis of the Effects of the Program

Results of a Box’s *M* test did not show homogeneity of the variance-covariance matrix for the global average academic performance of the five subjects’ scores used in the research. The value of The Box’s *M* test was significant (*F* = 3.02; *gl* = 1777031.77; *p* = 0.03). The violation of this assumption has a minimum effect if the groups are approximately equal in size (Hair et al., 1999). Because there are only two levels, sphericity is assumed.

To evaluate the impact of the program on academic performance, the scores obtained by both groups were compared before and after the intervention. As can be seen in Table 1, the interaction between the evaluation time (pre-test and post-test) and the program is significant (*p* ≤ 0.05).

**TABLE 1:** Summary of intra-inter subject univariate analysis of variance: Academic Performance.

	Font	Type III	*gl*	*F*	Significant	η^2^ partial	Observed power
**Academic Performance**	Intra	1.08	1	4.61	0.03	0.05	0.56
	Intra × Inter	1.44	1	6.14	0.01	0.07	0.69
	Error Intra	19.71	84	0.23			
	Inter	0.16	1	0.05	0.82	0.00	0.05
	Error Inter	274.82	84				

**FIGURE 1:**
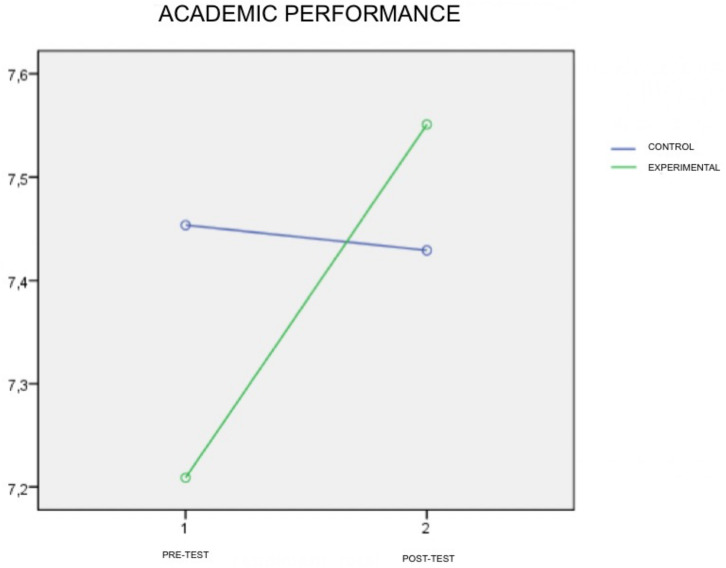
Academic Performance scores of the experimental and control groups at the pretest (1) and posttest (2) times.

There are significant differences between the students’ average scores. Students in the experimental group slightly improve their academic performance once the intervention program has been completed. Nevertheless, the observed power (testing the null hypothesis of no effect) doesn’t have a high value (0.69), so the size of the effect (η^2^) is not as desired. The differences in the averages of the scores can be seen in the Figure 1.

**FIGURE 2:**
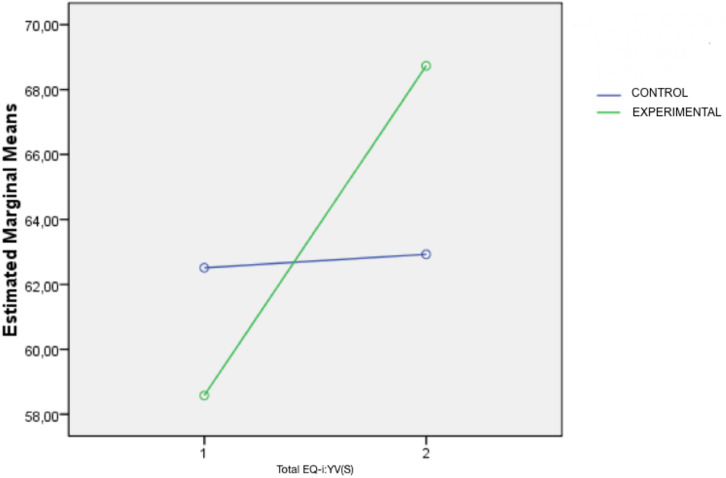
Total EQ-i: YV(S) scores of the experimental and control groups at the pretest (1) and posttest (2) times.

To evaluate the effectiveness of the program in students’ emotional competence, a GLM of repeated measures was also used. The effects of the intra-subject and inter-subject show that the effect of the interaction between the time of the evaluation (pre-test and post-test) and the implementation of the program is significant (*p* ≤ 0.05) (Table 2). There are significant differences between the students’ scores in the EQ-i: YV (S) as can be seen in Figure 2. However, the effect of the size (η^2^) is not as high as desired, maybe it is because of the small size of the sample.

**TABLE 2:** Summary of intra-inter subject univariate analysis of variance: Total EQ-i:YV(S).

	Font	Type III	*gl*	*F*	Significant	η^2^ partial	Observed power
**Total EQ-i:YV(S)**	Intra	486.87	1	12.50	0.00	0.13	0.94
	Intra × Inter	184.89	1	4.75	0.03	0.05	0.57
	Error Intra	3270.79	84				
	Inter	37.59	1	0.59	0.44	0.00	0.12
	Error Inter	5314.87	84				

## Discussion

During the past two decades the interest in social-emotional learning (SEL) has increased exponentially. Schools and teachers are aware of the importance of social-emotional competence in students’ cognitive development and their link to academic outcomes (Durlak et al., 2011). Despite that, SEL is not fully incorporated in every school. The lack of time and resources and the increasing demands on schools in terms of their requirements (diversity, inclusion, etc.) on a daily basis present an obstacle which is difficult to overcome.

According to Jones et al. (2017) in their review of effective SEL programs, it is not unusual that some schools, after implementing a SEL program, do not see the results they expected based on previous research. This could be due to the superficial implementation of the program in the school without being part of the school life.

OKAPI program has been designed to be integrated into the classes’ routines, to be taught using the existing curriculum (Bear et al., 2017) and with an approach that is positive and sensitive to the school environment. The purpose of the program was to be a unique tool to help teachers to improve class climate, student well-being, social competence, and academic performance.

Several studies have found that SEL programs have better results if they are conducted by teachers (Durlak et al., 2011). That’s why the design of the program, activities and strategies used were created as purposefully uncomplicated to facilitate their implementation.

The results of the study provide preliminary data supporting the effectiveness of the OKAPI program on students’ academic performance and emotional competence. The profile graphs show the differences obtained by the groups (experimental and control) in the pre-test and post-test situations, allowing us to visualize the meaning of the differences in favor of the experimental group in the variables studied. In reference to academic performance, the students’ average scores have risen in the experimental group from 7.2 points in the pre-test to almost 7.6 points on the post-test. It is a small improvement on the overall scores but it shows a tendency. In every subject evaluated the experimental group has increased their scores.

The OKAPI program uses different strategies that have been associated with different studies to an improvement of academic performance such as the use of cooperative learning methodology (Johnson et al., 2014; Santos et al., 2018).

Cooperative learning offers a simple method to facilitate the daily SEL practice. When students work together they improve their social competence through the interaction with their peers. At the same time they perform positive personal relationships and live the experience of building their social emotional competence in real situations. We believe this supports the creation of warm and supportive ties which are linked to better school performance (Osher et al., 2018). Moreover, research points out that cooperative learning increases students’ motivation and levels of self-esteem and self-efficacy (Johnson et al., 2014).

Several researchers have worked in programs with both social-emotional and school climate approaches which had a positive impact on school achievement (Cohen et al., 2009). The school and classroom environment has a role in shaping students’ emotions and influences school performance (Divecha and Bracket, 2020). The circumstances in which students’ learning take place matter (Suàrez et al., 2011). We agree with other studies that highlight the importance of developing a caring and supportive learning community in school (Issen, 2008). Strategies such as the Start Circle (the daily classroom meetings) or the different techniques used in the program to solve problems and manage their own emotions contribute to this objective.

Schools traditionally establish norms, lists of undesirable behaviors and consequences (Divecha and Bracket, 2020). To develop students’ emotions we need to go beyond that and offer strategies to help them to transform those undesirable behaviors for others that will help the construction of a safe and positive environment.

This research analyses the impact of an emotional education program, that has been created with a positive classroom climate approach, on academic performance. Although it uses direct instruction to teach emotional competences, the main elements of the program are integrated during the routines of the school day. This aspect is essential to develop emotional competences and to facilitate the implementation of the program. The OKAPI program is aligned with research that highlights that emotional education programs should be integrated from a school-wide perspective and in collaboration with positive school climate initiatives (Jones et al., 2017).

Although the results of the program’s effectiveness are positive, the effect size was relatively small. Further research is needed to determine the longer-term effect and it is also desirable to test it in larger samples. On the other hand, it would be interesting to use a standard test in order to obtain standardized date to the improvement in students’ performance.

## Limitations

This research has various limitations, including: the exclusive use of self-reporting as the method for collecting data (such methods are not very robust against possible bias in responses introduced by the subjects themselves), the impossibility of an accurate control over the variables in real situations in primary classrooms or the size of the sample used. Moreover, the participation of only two schools limits the extrapolation of results to other settings.

## Data Availability Statement

The datasets generated for this study are available on request to the corresponding author.

## Ethics Statement

The studies involving human participants were reviewed and approved by the University of Alicante Ethics Committee (Ref. UA-2015-07-06). Written informed consent to participate in this study was provided by the participants’ legal guardian/next of kin.

## Author Contributions

M-JM-G carried out a theoretical review of the topic, did fieldwork, program implementation, and worked on quantitative methods. RG-C was director of the research and carried out the quantitative methods.

## Conflict of Interest

The authors declare that the research was conducted in the absence of any commercial or financial relationships that could be construed as a potential conflict of interest.
